# Prognostic implications of late gadolinium enhancement at the right ventricular insertion point in patients with non-ischemic dilated cardiomyopathy: A multicenter retrospective cohort study

**DOI:** 10.1371/journal.pone.0208100

**Published:** 2018-11-28

**Authors:** Jeong-Eun Yi, Junbeom Park, Hye-Jeong Lee, Dong Geum Shin, Yookyung Kim, Minsuk Kim, Kihwan Kwon, Wook Bum Pyun, Young Jin Kim, Boyoung Joung

**Affiliations:** 1 Department of Cardiology, Ewha Womans University College of Medicine, Seoul, Republic of Korea; 2 Department of Radiology, Research Institute of Radiological Science, Yonsei University College of Medicine, Seoul, Korea; 3 Yonsei University Health System, Yonsei Cardiovascular Hospital, Yonsei University College of Medicine, Seoul, Republic of Korea; 4 Department of Radiology, College of Medicine, Ewha Womans University School of Medicine, Seoul, Republic of Korea; 5 Department of Pharmacology, School of Medicine, Ewha Womans University, Seoul, Republic of Korea; Ospedale del Cuore G Pasquinucci Fondazione Toscana Gabriele Monasterio di Massa, ITALY

## Abstract

**Introduction:**

The presence of late gadolinium enhancement (LGE) at the right ventricular insertion point (RVIP) on cardiac magnetic resonance (CMR) is generally believed to be nonspecific, but the clinical implication of this unique LGE pattern in patients with non-ischemic dilated cardiomyopathy (NICM) has not been elucidated.

**Objectives:**

We investigated the prognostic significance of RVIP-LGE in NICM patients.

**Methods:**

A total of 360 consecutive NICM patients referred for CMR (102 with no LGE, 50 with RVIP-LGE, 121 with left ventricular [LV]-LGE, and 87 with both an LV and RVIP-LGE) were studied. The primary endpoint was a composite of the all-cause death, hospitalization due to worsening of heart failure, and major arrhythmic events.

**Results:**

During a mean follow-up of 45.2 ± 36.5 months, 149 (41.4%) patients (22 [21.6%] no LGE vs. 16 [32.0%] RVIP-LGE vs. 62 [51.2%] LV-LGE vs. 49 [56.3%] both LV and RVIP-LGE, *P* < 0.0001) reached the primary endpoint. A Kaplan Meier curve demonstrated that RVIP-LGE patients had an intermediate trend of an event free survival rate for the composite endpoint (log-rank *P* < 0.0001). In a multivariable Cox regression model, LV-LGE (*P* = 0.008) and both LV and RVIP-LGE (*P* = 0.003) were significantly associated with a worse outcome, whereas RVIP-LGE was not (*P* = 0.101). In addition, RVIP-LGE patients (n = 32) had a more favorable outcome compared to LV-LGE patients (n = 32) even after matching the extent of the LGE (median 3.4% [interquartile range, 3.1–3.8], 8 [25.0%] RVIP-LGE vs. 20 [62.5%] LV-LGE, *P* = 0.002).

**Conclusions:**

LGE confined to the RVIP among NICM patients did not significantly increase the risk of adverse cardiac events, and also showed a better outcome than the same extent of LGE located in the LV. Identification of this unique LGE distribution may help refine the current risk stratification.

## Introduction

Non-ischemic dilated cardiomyopathy (NICM) is a primary myocardial disease occurring in approximately one-third of heart failure (HF) patients and is associated with significant morbidity and mortality [1]. Cardiac magnetic resonance (CMR) imaging with late gadolinium enhancement (LGE) has emerged as a promising, noninvasive tool for the detection and quantification of myocardial fibrosis that is frequently found in patients with NICM [2]. Recently, CMR studies in NICM patients have demonstrated that the presence of LGE strongly predicts poor clinical outcomes such as hospitalization for HF, fatal ventricular arrhythmias, and death [3,4].

LGE confined to the anterior and/or posterior right ventricular insertion points (RVIPs) (RVIP-LGE) are commonly seen in patients with hypertrophic cardiomyopathy (HCM), which is associated with a relatively better prognosis [5–7]. Previously, this focal LGE region has shown myocardial disarray or interstitial fibrosis rather than replacement fibrosis [7,8]. However, LGE at the RVIP is also found in other medical conditions and its clinical impact still remains uncertain [9–12]. To the best of our knowledge, there is a paucity of data concerning the role of LGE at the RVIP in the risk stratification of NICM for adverse cardiac events. Hence, the aims of this study were first to investigate the characteristics and prognostic significance of LGE confined to the RVIP, and second to identify the determinants of this unique LGE distribution in patients with NICM.

## Methods

### Study population

Between May 2003 and February 2016, 430 consecutive patients with newly diagnosed NICM referred for CMR at two tertiary hospitals in South Korea were retrospectively identified. All patients underwent CMR just after the diagnosis of NICM that was confirmed by clinical assessment, echocardiography and coronary angiography. NICM was defined according to the World Health Organization/International Society and Federation of Cardiology criteria [13]. Patients with symptoms or signs of heart failure, a reduced left ventricular ejection fraction (LVEF < 50%) without regional wall motion abnormalities, an increased LV end-diastolic diameter (LVEDD > 55mm), no prior history of a myocardial infarction or revascularization, and absence of significant coronary artery disease on coronary angiography (obstruction > 50% of 2 or more epicardial vessels or the left main or proximal left anterior descending coronary artery) [14] were included in this study. We excluded patients with acute myocarditis, severe valvular heart disease, hypertrophic cardiomyopathy, infiltrative cardiomyopathy or other specific cardiomyopathies and congenital heart disease. Forty patients who had contraindications to CMR and gadolinium-based contrast agents or that withdrew their consent for participation in the study were excluded.

The study protocol conformed to the principles outlined in the Declaration of Helsinki and was approved by the local ethics committee (EUMC 2017-09-019-004). All data were fully anonymized before access and the requirement for informed consent was waived due to the retrospective nature of this study. The names of ethics committees are as follows: Ewha Womans Mokdong Hospital and Severance Cardiovascular Hospital.

### Electrocardiogram and echocardiography

On the day of the CMR study, standard resting 12-lead electrocardiograms (ECGs) were obtained at a paper speed of 25 mm/s with a calibration of 10 mm/mV (GE Healthcare, Marquette, MAC 5500, Waukesha, WI). All ECGs were analyzed by 1 experienced reader who was blinded to the clinical data of the patient. We documented the rhythm and measured the heart rate (ventricular rate), PR interval, and QRS duration according to the established guidelines. The corrected QT (QTc) interval was automatically calculated using Bazett’s formula (QTc_Baz_ = $\mathrm{QT}/\sqrt{RR}$) and a prolonged QTc was defined as a QTc interval of >440ms [15]. Transthoracic Doppler echocardiography was performed within one week of the CMR and all measurements were obtained as by the recommended standard guidelines [16].

### CMR acquisition and analysis

All patients underwent CMR studies on a 1.5 Tesla whole-body scanners (InteraAchieva, Phillips Medical System, Best, The Netherlands or Philips Healthcare, Andover, MA, USA) with a phase array cardiac coli and the same CMR protocol was adopted in both centers. Electrocardiogram (ECG)-gated cine images were acquired in the horizontal, vertical, and short-axis views using a breath-hold cine steady-state, free precession sequence (field of view, 360 ⅹ360 mm; matrix, 256 ⅹ 256 mm; slice thickness, 8 mm; TR [repetition time]/TE [echo time], 3.4/1.7 ms; flip angle, 50°; 25 frames for the cardiac cycle). A stack of contiguous short-axis slices from the base to apex were obtained without an interslice gap. Late enhancement images were acquired 10 minutes after infusing intravenous gadolinium-DTPA (0.2mmol/kg; gadoteratedimeglumine; Dotarem, Geurbet) using a T1-weighted 2-dimensional gradient echo inversion-recovery sequence (field of view, 360 ⅹ360 mm; matrix, 521 ⅹ 512 mm; slice thickness, 8 mm; TR/TE, 5.3/1.6 ms; flip angle, 15°; no interslice gap) in the same views used for the cine images. An ECG-synchronized image acquisition in the mid-diastole phase was performed in order to minimize any cardiac motion induced artifact. The inversion time (T1) was individually adapted to nullfy the signal of normal myocardium using a dedicated T1 determining sequence [17].

All CMR images were analyzed off-line using a dedicated software program (CMR42, Circle Cardiovascular Imaging, Calgary, Alberta, Canada) by the consensus of 2 experienced radiologists blinded to the patient clinical data and outcome. The left and right ventricular volumes and ejection fraction (EF) were measured from the cine images using a semi-automatic segmentation in the software and all volume measurements were normalized to the body surface area. The LGE was visually assessed for each segment using a modified American Heart Association (AHA) 16-segment left ventricular (LV) model [18] and considered to be present if there was a region of discernible high signal intensity upon visual inspection. The presence of LGE at the RVIP was defined as a focal, hyperintensity area confined to the junction of the RV wall into the anterior and/or posterior interventricular septum in at least two consecutive short-axis images. Factors affecting the image quality were checked, and the potential pitfalls and artifacts (e.g. blood pool, epi- and pericardial fat, partial volume effects, vessels, or ghosting artifacts) mimicking myocardial scar in the delayed enhancement CMR imaging were also excluded [19]. If LGE was detected, patterns were classified either as subendocardial, midwall, subepicardial, transmural or patchy [20]. The myocardial volume was obtained using a semiautomatic segmentation of the endocardial and epicardial borders in each short axis image. The LGE volume was quantified from a short-axis stack of images using a full width with a half maximum (FWHM) technique and the region of interest was drawn in the area of the maximum signal intensity of a visible LGE for the FWHM threshold. Both the myocardial and LGE volumes were estimated from the sum of each area for each slice, multiplied by the slice thickness. The extent of the LGE was expressed as a percentage of the LGE, which was derived by dividing the LGE volume by the myocardial volume, and the quotient multiplied by 100. The presence and extent of the LGE were evaluated twice by 2 independent expert readers who were blinded to all the patient details. The inter-observer agreement between the two readers in regard to the presence or absence of LGE was substantial (kappa value = 0.827, *P* < 0.005). The intra-observer variability of the LGE quantification for the coefficient of variation (CV) and intra-class correlation coefficient (ICC) were 12.5% and 0.99 (95% confidence interval [CI] 0.97–0.99), respectively. Also, there was good inter-observer agreement for RVIP-LGE (kappa value = 0.785, *P* < 0.005), and the intra-observer variability of RVIP-LGE quantification for the CV and ICC was 12.1% and 0.97 (95% CI 0.96–0.98), respectively.

### Clinical follow-up and outcome events

Patients underwent regular follow-up every 3 to 6 months via clinic visits or telephone interviews. The primary endpoint was a composite of the all-cause death and hospitalization due to worsening of heart failure (CHF) and major arrhythmic events. Hospitalization for HF was defined as the first readmission with worsening signs or symptoms of HF requiring treatment with i.v. therapies (i.e. diuretics, vasodilators, and inotropes), mechanical or surgical interventions, or initiation of ultrafiltration, hemofiltration or dialysis. Major arrhythmic events included sustained ventricular tachycardia (VT), ventricular fibrillation (VF), appropriate implantable cardioverter defibrillator (ICD) discharges, and sudden cardiac death (SCD). Sustained VT was defined as three or more consecutive ventricular extrasystoles of ≥100 beats/min lasting for ≥30 sec on the ECG or 24-h Holter monitoring or that requiring intervention for termination. VF was determined by QRS complexes with a markedly different morphology, axis, and amplitude, no identifiable P waves, and irregular ventricular rates of ≥300 beats/min. Appropriate ICD discharges were defined as a device shock or anti-tachycardia overdrive pacing for sustained VT based upon a programmed rate cutoff of the ICD (>180 beats/min) or VF. The cause and date of the death were identified using the information from the National Population Registry of Korea National Statistical Office as well as the medical records at the time of the death. SCD was defined as an unexpected death with or without documented ventricular arrhythmias occurring within 1h of the cardiac symptoms, or nocturnal death with no antecedent history of progressive cardiac deterioration.

### Statistical analysis

All statistical analyses were conducted using SPSS version 18.0 software (SPSS Inc., Chicago, Illinois, USA). Statistical significance was accepted for two-tailed values of *P* < 0.05. Continuous variables were expressed as the mean ± SD or median with a corresponding interquartile range (IQR) and categorical variables as the n (%). Comparisons of variables across groups were performed using a one-way ANOVA combined with a Bonferroni post hoc analysis or Kruskal-Wallis H test and Chi-square (χ^2^) or Fisher's exact test, as appropriate. Hazard ratios of the primary endpoint and its components were calculated with univariate Cox proportional hazard models with a computed 95% confidence interval (CI). The Kaplan-Meier method with log-rank tests was used to estimate the event free survival curves for each category of NICM. To control for any potential confounders, multivariate Cox proportional hazard models were built using covariates identified as significant in the univariate analysis or known to affect the primary endpoint. A post-hoc subgroup analysis was performed for patients with an LVEDVI less than the median of 160ml/m^2^. A multivariate binary logistic regression analysis was performed to assess the determinants of an LGE confined to the RVIP.

## Results

### Patient characteristics

**Fig 1:**
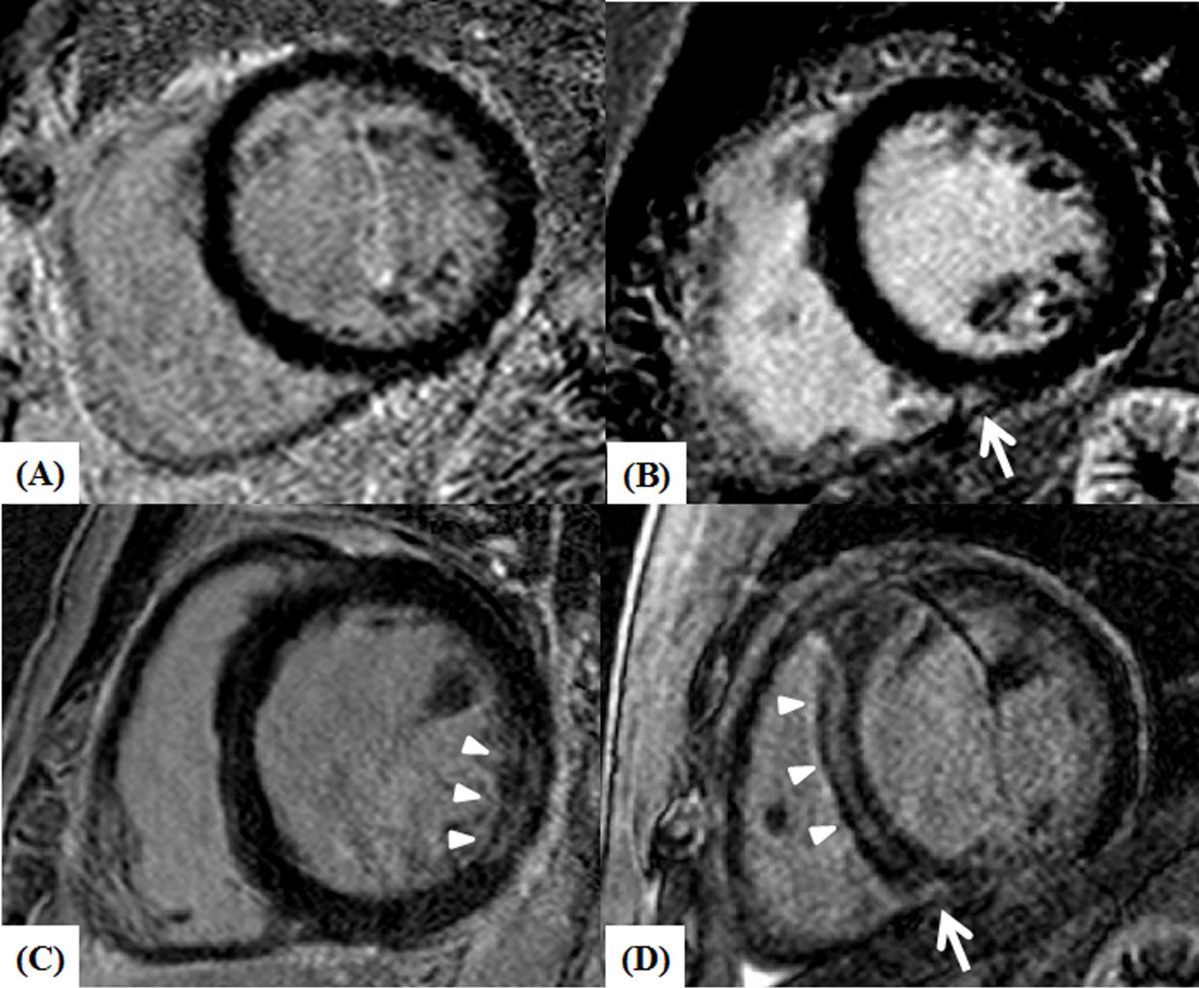
**Left ventricle short-axis CMR images of NICM patients showing no LGE (A), patchy LGE confined to the inferior RV insertion area (B, white arrow), midwall LGE at the inferolateral wall of LV (C, arrow heads), and LGE at the midwall of septum (arrow heads) and inferior RV insertion area (white arrow) (D).** CMR: cardiac magnetic resonance; NICM: non-ischemic dilated cardiomyopathy; LGE: late gadolinium enhancement; RV: right ventricular.

A total of 360 patients (36.9% female, mean age 54 ± 15 years) were enrolled and categorized into 4 groups according to the presence or absence and location of the LGE in the heart: no LGE (n = 102, 28.3%), RVIP-LGE (n = 50, 13.9%), LV-LGE (n = 121, 33.6%), and both LV and RVIP-LGE (n = 87, 24.2%) (Fig 1). The group baseline characteristics are summarized in Table 1. RVIP-LGE patients were younger and had higher systolic and diastolic blood pressure (BP) levels, but the gender distribution, BMI, and co-morbidity profiles did not significantly differ among the groups. Loop diuretics were more frequently used in the RVIP-LGE patients than those without LGE. Left bundle branch block (LBBB) was more common in the no LGE and RVIP-LGE patients than in the LV-LGE groups. RVIP-LGE patients had a more prolonged QTc interval, higher NT-proBNP level than the other groups, and greater E/E’ ratio than the no LGE group.

**Table 1:** Patients characteristics.

	Total (n = 360)	No LGE (n = 102)	RVIP-LGE (n = 50)	LV-LGE (+)		P Value
				RVIP-LGE (-) (n = 121)	RVIP-LGE (+) (n = 87)	
Age, y	54 ± 15	54 ± 15	50 ± 13	57 ± 15	53 ± 14	0.040
Female, n (%)	133 (36.9)	43 (42.2)	23 (46.0)	42 (34.7)	25 (28.7)	0.126
BMI, kg/m^2^	25 (22–27)	26 (22–27)	25 (21–27)	24 (21–25)	23 (22–28)	0.149
SBP, mmHg	113 (105–130)	120 (108–130)	120 (110–138)	112 (104–130)	112 (100–125)	0.045
DBP, mmHg	70 (68–80)	70 (69–80)	80 (70–86)	71 (66–80)	70 (60–80)	0.003
NYHA class ≥ III, n (%)	187 (51.9)	46 (45.1)	22 (44.0)	64 (52.9)	55 (63.2)	0.054
Diabetes mellitus, n (%)	107 (29.7)	34 (33.3)	10 (20.0)	38 (34.1)	25 (28.7)	0.376
Hypertension, n (%)	170 (47.2)	42 (41.2)	27 (54.0)	54 (44.6)	47 (54.0)	0.225
Smoker, n (%)	126 (35.1)	30 (29.4)	15 (30.0)	43 (35.5)	38 (43.7)	0.195
Alcohol, n (%)	137 (38.2)	39 (38.2)	14 (28.0)	44 (36.4)	40 (46.0)	0.228
Medications						
ACEi or ARB, n (%)	333 (92.5)	92 (90.2)	49 (98.0)	114 (94.2)	78 (89.7)	0.208
Beta-blocker, n (%)	270 (75.0)	76 (74.5)	39 (78.0)	90 (74.4)	65 (74.7)	0.963
Loop diuretics, n (%)	291 (80.8)	74 (72.5)	42 (84.0)	98 (81.0)	77 (88.5)	0.043
Spironolactone, n (%)	233 (64.7)	58 (56.9)	31 (62.0)	84 (69.4)	60 (69.0)	0.189
Digoxin, n (%)	127 (35.4)	28 (27.5)	16 (32.0)	47 (38.8)	36 (41.9)	0.153
AFib or AF, n (%)	67 (18.6)	23 (22.5)	5 (10.0)	28 (23.1)	11 (12.6)	0.066
LBBB, n (%)	55 (15.3)	25 (24.5)	10 (20.0)	11 (9.1)	9 (10.5)	0.005
PR interval, ms	168 (160–188)	174 (154–184)	171 (160–185)	168 (160–189)	176 (162–192)	0.626
QRS interval, ms	102 (93–117)	99 (92–116)	101 (92–131)	104 (96–116)	108 (94–123)	0.485
QTc interval, ms	469 ± 42	466 ± 43	482 ± 35	461 ± 40	473 ± 44	0.020
E/E’ ratio	18.8 ± 9.3	16.3 ± 7.6	19.6 ± 7.2	20.0 ± 10.6	19.3 ± 9.8	0.046
SPAP, mmHg	31 (25–48)	34 (24–45)	33 (24–44)	38 (26–49)	30 (27–51)	0.266
NT-proBNP, pg/ml	2034 (1038–5641)	1885 (464–4680)	3428 (1202–4783)	3156 (1404–6512)	2168 (1097–6936)	0.029
Troponin T, ng/ml	0.016 (0.010–0.026)	0.010 (0.010–0.030)	0.010 (0.010–0.020)	0.014 (0.010–0.025)	0.016 (0.010–0.052)	0.210
CMR characteristics						
LVEF, %	25 ± 9	27 ± 9	25 ± 9	25 ± 10	23 ± 7	0.035
LVEDVI, ml/m^2^	165.9 (132.2–196.7)	152.0 (124.7–179.4)	139.1 (126.4–173.0)	163.1 (130.8–199.4)	181.3 (144.0–235.2)	0.001
LVESVI, ml/m^2^	131.4 ± 54.3	115.9 ± 40.6	113.1 ± 41.0	132.7 ± 61.9	150.8 ± 55.0	0.001
RVEF, %	37.5 ± 14.8	40 ± 15	42 ± 17	37 ± 12	34 ± 16	0.064
RVEDVI, ml/m^2^	106.1 ± 43.2	98.0 ± 31.4	97.6 ± 36.4	110.8 ± 52.0	111.0 ± 43.8	0.326
RVESVI, ml/m^2^	71.0 ± 41.5	63.7 ± 29.2	60.6 ± 38.4	75.2 ± 47.9	76.7 ± 43.2	0.247
RVIP-LGE location						<0.0001
Anterior RVIP, n (%)	0 (0)	―	0 (0)	―	0 (0)	
Posterior RVIP, n (%)	93 (25.8)	―	47 (94.0)	―	46 (52.9)	
Both RVIPs, n (%)	41 (11.4)	―	3 (6.0)	―	38 (43.7)	
LGE pattern						
subepicardial, n (%)	9 (2.5)	―	0 (0)	5 (4.1)	4 (4.6)	0.400
midwall, n (%)	168 (46.7)	―	2 (4.0)	95 (78.5)	71 (81.6)	<0.0001
subendocardial, n (%)	13 (3.6)	―	0 (0)	11 (9.1)	2 (2.3)	0.019
transmural, n (%)	29 (8.1)	―	0 (0)	18 (14.9)	11 (12.6)	0.017
patchy, n (%)	122 (33.9)	―	49 (98.0)	13 (10.7)	60 (69.0)	<0.0001
LGE extent (%)	4.1 (0.0–7.3)	―	3.2 (2.8–3.5)	5.6 (3.9–10.0)	7.5 (4.3–26.1)	<0.0001

LGE: late gadolinium enhancement; RVIP: right ventricular insertion point; LV: left ventricular; BMI: body mass index; SBP: systolic blood pressure; DBP: diastolic blood pressure; NYHA: New York Heart Association; ACEi: angiotensin converting enzyme inhibitor; ARB: angiotensin receptor blocker; AFib: atrial fibrillation; AF: atrial flutter; LBBB: left bundle branch block; QTc: corrected QT; E/E’: the ratio of peak early diastolic transmitral flow velocity to averaged value of peak early diastolic septal and lateral mitral annular velocities; SPAP: systolic pulmonary arterial pressure; NT-proBNP: N-terminal pro-B-type natriuretic peptide; CMR: cardiac magnetic resonance; LVEF: left ventricular ejection fraction; LVEDVI: left ventricular end diastolic volume index; LVESVI: left ventricular end systolic volume index; RVEF: right ventricular ejection fraction; RVEDVI: right ventricular end diastolic volume index; RVESVI: right ventricular end systolic volume index.

The mean LVEF in the total study population was 25 ± 9%. RVIP-LGE patients had a similar LVEF as the LV-LGE patients, but had lower LV end-diastolic and end-systolic volume indices compared to other groups. Although the RVIP-LGE patients tended to have a higher RVEF, there were no statistically significant differences in the RV end-diastolic and end-systolic volume indices between the groups. LGE at only the posterior RVIP was more common in the RVIP-LGE patients, whereas LGE at both RVIPs was more frequently observed in the patients with LGE in both the LV and RVIP. LGE at only the anterior RVIP was not found in either group. Patchy pattern of LGE was predominantly found more often in the RVIP-LGE patients than other groups and the extent of the LGE was greatest in the patients with LGE at both the LV and RVIP.

### Clinical outcome

**Fig 2:**
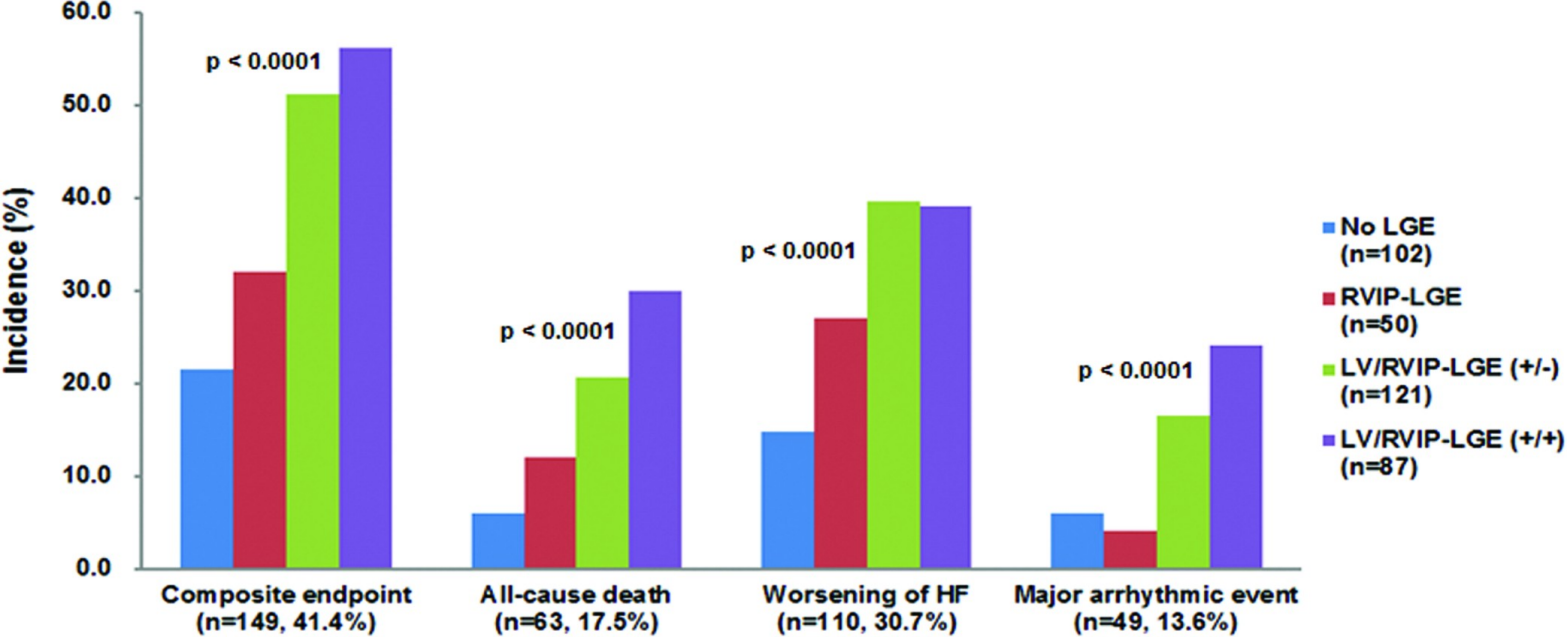
Clinical outcomes during the follow-up in patients with no LGE (n = 102), RVIP-LGE (n = 50), LV-LGE (n = 121), and both LV and RVIP-LGE (n = 87). LGE: late gadolinium enhancement; RVIP: right ventricular insertion point; LV: left ventricular.

**Fig 3:**
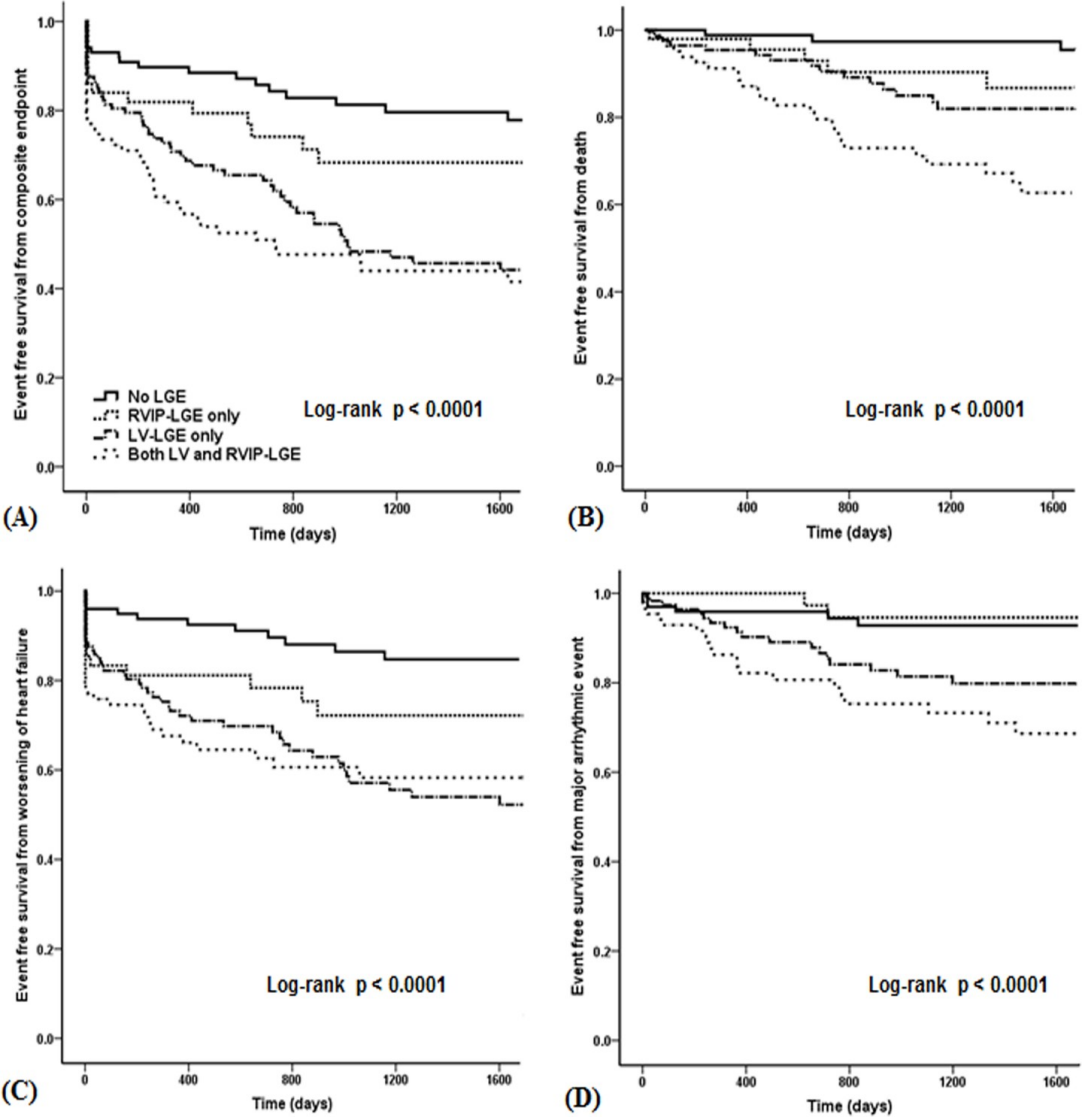
**Kaplan-Meier event-free survival curves** for the (A) composite endpoint, (B) all-cause death, (C) worsening of heart failure, (D) major arrhythmic events among the 4 groups stratified according to the presence and location of the LGE.

During a mean follow-up duration of 45.2 ± 36.5 months, 149 (41.4%) patients reached the primary endpoint. Thereof 63 (17.5%) patients died, 110 (30.7%) were hospitalized due to worsening of HF and 49 (13.6%) suffered major arrhythmic events. Overall, the composite endpoint most commonly occurred in patients with an LGE in both the LV and RVIP, followed by the LV-LGE patients, RVIP-LGE patients, and lowest in the patients without LGE. Similar results were observed for the all-cause death, but the incidence of worsening HF did not differ between the LV-LGE patients with and without RVIP-LGE. Major arrhythmic events least frequently occurred in the RVIP-LGE patients and none of them underwent implantable cardioverter defibrillation (ICD) implantations for primary or secondary prevention (Fig 2). The Kaplan-Meier survival curves significantly differed among the groups. RVIP-LGE patients exhibited an intermediate trend of an event free survival rate for the composite endpoint (Fig 3A, log-rank *P* < 0.0001), all-cause death (Fig 3B, log-rank *P* < 0.0001), and worsening of HF (Fig 3C, log-rank *P* < 0.0001). For the major arrhythmic events, the RVIP-LGE patients had a higher event free survival rate than the LV-LGE patients with or without RVIP-LGE, which was similar to that of the patients without LGE (Fig 3D, log-rank *P* < 0.0001).

**Fig 4:**
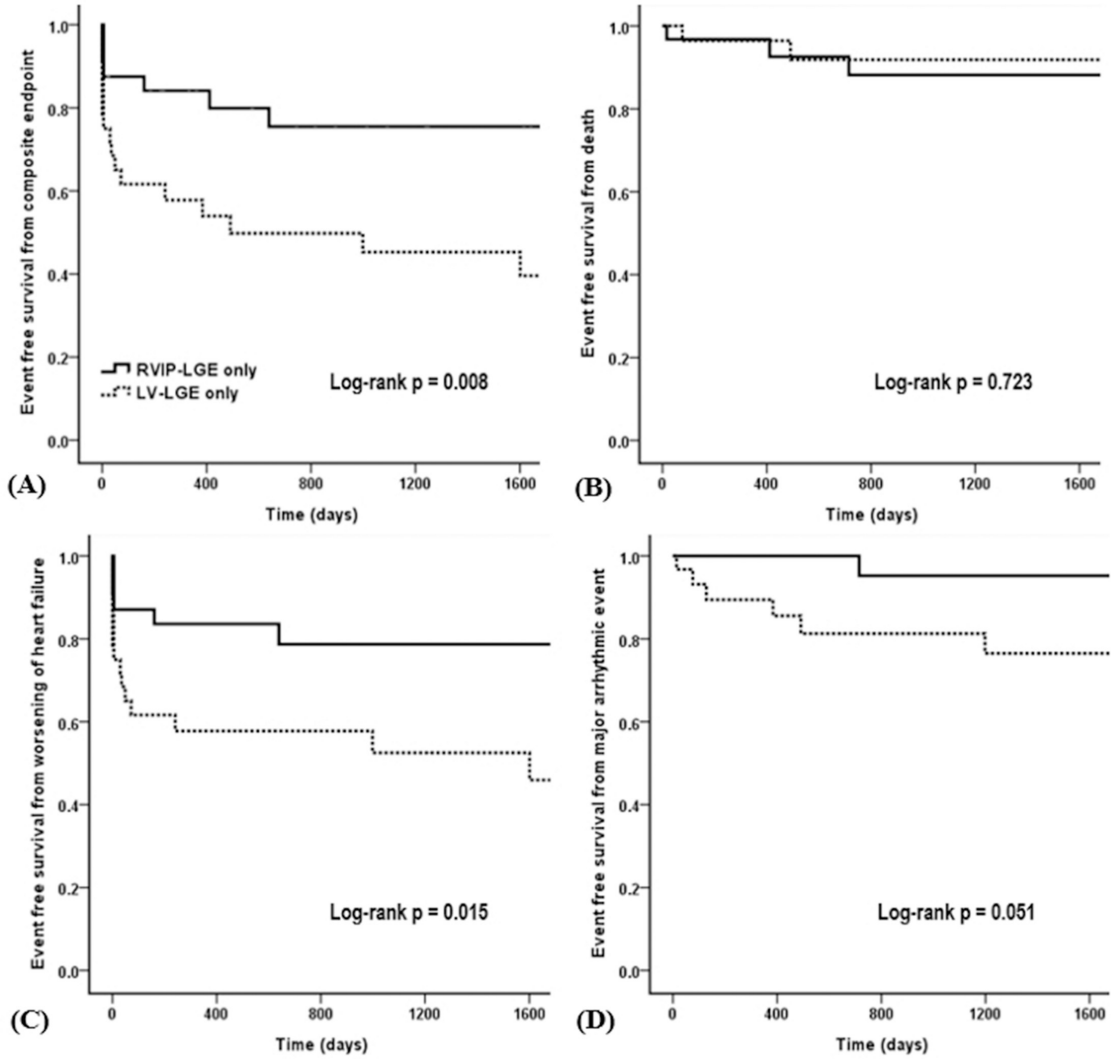
**Kaplan-Meier event-free survival curves** for the (A) composite endpoint, (B) all-cause death, (C) worsening of heart failure, (D) major arrhythmic events in the LGE extent matched RVIP-LGE (n = 32) and LV-LGE (n = 32) groups. LGE: late gadolinium enhancement; RVIP: right ventricular insertion point; LV: left ventricular.

A multivariate Cox regression analysis demonstrated that the presence of LGE at only the RVIP did not significantly increase the risk of adverse outcomes compared with the absence of an LGE (Table 2). Additionally, we compared the clinical outcomes between the patients with LGE isolated to the RVIP (n = 32) and those with LV-LGE only (n = 32) after matching the extent of the LGE to examine if the favorable outcome of the RVIP-LGE was related to the small LGE size, rather than the location of the LGE. Although the LGE extent was same (median 3.4% [interquartile range, 3.1–3.8]) in both groups (Table 3), RVIP-LGE patients had a significantly lower incidence of the composite endpoint and worsening HF than the LV-LGE patients (Table 4). Kaplan-Meier curves for the composite endpoint and worsening HF also demonstrated significant differences between them (Fig 4).

**Table 2:** Cox proportional hazard analysis for the predictors of a composite clinical outcome in the total study population (n = 360).

	Univariate analysis		Multivariate analysis (χ2 = 40.999, p < 0.0001)	
	HR (95% CI)	*P* Value	HR (95% CI)	*P* Value
LGE location				
No LGE	1 (reference)		1 (reference)	
RVIP-LGE	1.629 (0.855–3.102)	0.138	3.168 (0.800–12.541)	0.101
LV-LGE	3.022 (1.854–4.924)	<0.0001	5.742 (1.564–21.080)	0.008
LV and RVIP-LGE	3.680 (2.219–6.104)	<0.0001	7.158 (1.963–26.107)	0.003
LGE extent (%)	1.035 (1.024–1.045)	<0.0001	1.023 (0.999–1.047)	0.060
Age, y	1.007 (0.995–1.019)	0.258	1.019 (0.997–1.041)	0.085
Female	0.958 (0.687–1.336)	0.802	1.930 (0.905–4.115)	0.089
NYHA class ≥ III	1.143 (0.828–1.579)	0.416	0.810 (0.456–1.437)	0.471
Diabetes mellitus	1.100 (0.785–1.541)	0.580	1.669 (0.946–2.943)	0.077
Smoking	1.259 (0.906–1.750)	0.170	1.248 (0.576–2.700)	0.574
Ln NT-proBNP, pg/ml	1.243 (1.078–1.433)	0.003	0.911 (0.705–1.178)	0.478
LVEF, %	0.981 (0.962–1.001)	0.061	0.972 (0.920–1.027)	0.318
LVEDVI, ml/m^2^	1.004 (1.002–1.007)	0.002	1.001 (0.995–1.006)	0.819
RVEF, %	0.990 (0.977–1.004)	0.160	1.015 (0.983–1.048)	0.366
RVEDVI, ml/m^2^	1.008 (1.001–1.014)	0.021	1.004 (0.997–1.012)	0.271

HR: hazard ratio; CI: confidence interval; LGE: late gadolinium enhancement; RVIP: right ventricular insertion point; NYHA: New York Heart Association; Ln NT-proBNP: log transformed N-terminal pro-B-type natriuretic peptide; LVEF: left ventricular ejection fraction; LVEDVI: left ventricular end-diastolic volume index; RVEF: right ventricular ejection fraction; RVEDVI: right ventricular end diastolic volume index.

**Table 3:** Comparison of the baseline characteristics between the RVIP-LGE (n = 32) and LV-LGE (n = 32) groups after matching the extent of the LGE.

Variables	RVIP-LGE (n = 32)	LV-LGE (n = 32)	*P* Value
Age, y	50±12	56±14	0.059
Male, n (%)	18 (56.3)	21 (65.6)	0.442
BMI, kg/m^2^	25 (21–27)	25 (23–26)	0.582
SBP, mmHg	123±21	118±22	0.387
DBP, mmHg	80±14	72±14	0.030
NYHA class ≥ III, n (%)	14 (43.8)	17 (53.1)	0.453
Diabetes mellitus, n (%)	6 (18.8)	13 (40.6)	0.055
Hypertension, n (%)	18 (56.3)	16 (50.0)	0.616
Smoker, n (%)	11 (31.4)	12 (37.5)	0.794
Alcohol, n (%)	10 (31.3)	14 (43.8)	0.302
Medications			
ACEi or ARB, n (%)	32 (100.0)	30 (93.8)	0.492
Beta-blocker, n (%)	26 (81.3)	21 (65.6)	0.157
Loop diuretics, n (%)	26 (81.3)	27 (84.4)	0.740
Spironolactone, n (%)	20 (62.5)	26 (81.3)	0.095
Digoxin, n (%)	9 (28.1)	14 (43.8)	0.193
Electrocardiographic findings			
AFib or AF, n (%)	1 (3.1)	12 (37.5)	0.001
LBBB, n (%)	6 (18.8)	6 (18.8)	1.000
PR interval (msec)	174±17	179±25	0.430
QRS interval (msec)	115±30	109±27	0.430
QTc interval (msec)	486±34	453±32	<0.0001
Echocardiographic parameters			
E/E’ ratio	20.1±8.5	19.8±12.4	0.923
SPAP, mmHg	38±14	37±14	0.834
Laboratory findings			
NT-proBNP, pg/ml	3658 (1202–4783)	1919 (1002–3856)	0.379
Troponin T, ng/ml	0.011 (0.010–0.021)	0.011 (0.301–0.019)	0.873
CMR findings			
LVEF, %	25±9	26±11	0.612
LVEDVI, ml/m^2^	156±48	152±46	0.827
LVESVI, ml/m^2^	113.1 (84.0–147.9)	93.3 (78.8–164.3)	0.868
RVEF, %	43±17	38±11	0.229
RVEDVI, ml/m^2^	98.2 (69.9–111.7)	93.2 (69.8–100.7)	0.757
RVESVI, ml/m^2^	48.2 (34.9–70.4)	48.7 (40.1–79.7)	0.723
LGE extent, %	3.4 (3.1–3.8)	3.4 (3.1–3.8)	0.973

RVIP: right ventricular insertion point; LV: left ventricular; LGE: late gadolinium enhancement; BMI: body mass index; SBP: systolic blood pressure; DBP: diastolic blood pressure; NYHA: New York Heart Association; ACEi: angiotensin converting enzyme inhibitor; ARB: angiotensin receptor blocker; AFib: atrial fibrillation; AF: atrial flutter; LBBB: left bundle branch block; QTc: corrected QT; E/E’: the ratio of peak early diastolic transmitral flow velocity to averaged value of peak early diastolic septal and lateral mitral annular velocities; SPAP: systolic pulmonary arterial pressure; NT-proBNP: N-terminal pro-B-type natriuretic peptide; CMR: cardiac magnetic resonance; LVEF: left ventricular ejection fraction; LVEDVI: left ventricular end diastolic volume index; LVESVI: left ventricular end systolic volume index; RVEF: right ventricular ejection fraction; RVEDVI: right ventricular end diastolic volume index; RVESVI: right ventricular end systolic volume index.

**Table 4:** Comparison of the clinical outcomes in the RVIP-LGE (n = 32) and LV-LGE (n = 32) groups after matching the extent of the LGE.

Outcome measure, n (%)	RVIP-LGE (n = 32)	LV-LGE (n = 32)	*P* Value
Composite endpoint	8 (25.0)	20 (62.5)	0.002
All-cause death	4 (12.5)	7 (21.9)	0.320
Worsening of heart failure	6 (9.4)	17 (53.1)	0.005
Major arrhythmic event	1 (3.1)	6 (18.8)	0.104
ICD implantation	0 (0)	3 (9.4)	0.238

RVIP: right ventricular insertion point; LGE: late gadolinium enhancement; LV: left ventricular; ICD: implantable cardioverter defibrillator

### Determinants of an LGE confined to the RVIP

In the multivariate logistic regression analysis, the age, a female gender, diastolic BP, presence of LBBB, and a prolonged QTc interval (>440msec) were independently associated with LGE at only the RVIP (Table 5).

**Table 5:** Determinants of an LGE confined to the RVIP.

Variables	Univariate analysis		Multivariate analysis (χ2 = 34.601, p < 0.0001)	
	OR (95% CI)	*P* Value	OR (95% CI)	*P* Value
Age, y	0.90 (0.960 ― 0.999)	0.043	0.954 (0.921 ― 0.988)	0.008
Female	1.549 (0.848 ― 2.830)	0.155	6.264 (1.836 ― 21.377)	0.003
Diastolic BP, mmHg	1.036 (1.013 ― 1.060)	0.002	1.055 (1.015 ― 1.096)	0.006
Diabetes mellitus	0.549 (0.564 ― 1.143)	0.109	0.859 (0.279 ― 2.644)	0.791
Smoker	0.791 (0.413 ― 1.516)	0.480	0.856 (0.227 ― 3.232)	0.818
LBBB	1.467 (0.685 ― 3.141)	0.324	6.233 (1.614 ― 24.062)	0.008
QTc interval > 440 msec	4.560 (1.579 ― 13.166)	0.005	5.490 (1.168 ― 25.810)	0.031
LVEF, %	1.001 (0.963 ― 1.041)	0.948	0.982 (0.927 ― 1.040)	0.527
E/E’ ratio	1.011 (0.977 ― 1.045)	0.538	1.016 (0.956 ― 1.080)	0.616
SPAP, mmHg	0.990 (0.968 ― 1.013)	0.404	0.966 (0.922 ― 1.012)	0.140

LGE: late gadolinium enhancement; RVIP: right ventricular insertion point; OR: odds ratio; CI: confidence interval; BP: blood pressure; LBBB: left bundle branch block; QTc: corrected QT; LVEF: left ventricular ejection fraction; E/E’: the ratio of the peak early diastolic transmitral flow velocity to the averaged value of the peak early diastolic septal and lateral mitral annular velocities; SPAP: systolic pulmonary artery pressure.

## Discussion

The main findings of this study among the patients with NICM were as follows. First, the presence of LGE at only the RVIP did not significantly increase the risk of adverse cardiac events. Second, a young age, female gender, elevated diastolic BP, presence of LBBB, and prolonged QTc interval were independent predictors of LGE confined to the RVIP. To the best of our knowledge, this report is the first study to evaluate the prognostic value of the LGE at the RVIP in NICM patients.

Although the presence of LGE has been described as a poor prognostic indicator in patients with NICM [3,4], only a few studies have mentioned the clinical significance of LGE at the RVIP [21,22]. Neilan T et al. found in a study of 162 NICM patients that a focal LGE at the RVIP was not associated with an increased risk of adverse outcomes [21]. On the contrary, data from Gaztanaga J et al. showed a worse outcome in patients with RVIP-LGE than in those with various patterns of LV-LGE [22]. However, the aforementioned studies did not focus particularly on LGE confined to the RVIP and accordingly, had a relatively small sample size and limited number of events in the patients with RVIP-LGE. In the present study, we demonstrated a more favorable outcome in patients with LGE at only the RVIP than in those with LV-LGE, but at the same time, it was less favorable than that in patients without LGE.

Myocardial fibrosis is one of the most common histopathologic findings of the failing heart, which is characterized by a progressive accumulation of collagen components leading to an increased myocardial stiffness, cardiac remodeling, and worsening ventricular systolic function [23]. CMR imaging with LGE (CMR-LGE) allows a sensitive and reproducible assessment of replacement myocardial fibrosis, but does not detect interstitial fibrosis, which is another subtype of myocardial fibrosis in dilated cardiomyopathy (DCM) [24]. In our study, despite a small amount of myocardial scar, the RVIP-LGE group had an elevated LV filling pressure comparable to that of the LV-LGE groups and the use of loop diuretics was more common in the RVIP-LGE patients than in those without LGE. Furthermore, the RVIP-LGE group had a significantly longer QTc interval than the other groups, and a prolonged QTc interval was an independent predictor of LGE confined to the RVIP. Recently, Malaty AN et al. demonstrated a higher LV filling pressure in DCM patients without LGE than in those with LGE, postulating a causal role of interstitial fibrosis in increased LV stiffness [25]. The QTc interval has been also reported as an electrocardiographic parameter associated with LV diastolic dysfunction [26]. Indeed, in a study of 241 HF patients, a prolonged QTc interval was a strong predictor of death due to worsening HF [15]. Considering that the LV diastolic, rather than systolic function is closely related to exercise tolerance [27], LGE confined to the RVIP could be an important surrogate marker reflecting increased LV stiffness beyond the extent of the LGE. Interestingly, in a post-hoc subgroup analysis of patients with a less dilated LV cavity (LVEDVI ≤ the median value of 160ml/m^2^), we found that the RVIP-LGE patients had a greater risk of experiencing the composite endpoint than the patients without LGE (adjusted HR, 4.20; 95% CI, 1.21–14.52, *P* = 0.024), and had a similar risk as the LV-LGE patients with and without RVIP-LGE (S1 Table). The Kaplan-Meier analysis also indicated that the RVIP-LGE patients were at a significantly increased risk for adverse outcomes as compared to the patients without LGE during the follow-up (S1 Fig, log-rank *P* = 0.003). These results suggested that the identification of this unique LGE distribution may be useful in the prognostic stratification from the early stages of NICM [28]. On the other hand, in patients with LV myocardial scar on the CMR-LGE, the additional clinical impact of an RVIP-LGE was not significant (Fig 3A, *P* = 0.571).

Although the myocardial scar observed by CMR-LGE is thought to be a potential substrate for re-entrant circuits [29], we found no significant difference in the occurrence of major arrhythmic events between the patients with RVIP-LGE and those without LGE. Previous studies documented that the characteristics of the LGE, including the pattern and extent, are associated with the risk of arrhythmic events. For instance, Assomull RG et al. and Gulati A et al. showed a strong association between midwall fibrosis and SCD or ventricular arrhythmias in patients with NICM [3,4]. In a recently published study, the extent of the myocardial scar was an independent predictor of arrhythmic events [21]. However, in our study, most patients with RVIP-LGE demonstrated a patchy pattern of the LGE, and a midwall pattern was observed in only 2 (4.0%) patients. Moreover, the extent of the LGE was significantly lower in the patients with RVIP-LGE than those with LV-LGE. These CMR characteristics could explain why patients with LGE confined to the RVIP did not have a significant arrhythmic risk compared to the patients without LGE in this study.

In the present study, we found an independent association between RVIP-LGE and LBBB, which was a consistent finding of the previous reports that described the paradoxical motion of the interventricular septum as a major determinant of LGE at the VIP [9]. Also, LGE at the RVIP was related to a young age, female gender, elevated diastolic BP, and prolonged QTc interval. However, our data showed no significant relationship between RVIP-LGE and SPAP, despite the involvement of the RVIP has been reported as a typical pattern of LGE in pulmonary hypertension. This seems to be attributed to our population characteristics with a relatively lower proportion and lesser degree of pulmonary hypertension (no [60.7%], mild [28.0%], moderate [11.3%], severe [0%]), compared with previously published studies of patients with pulmonary hypertension [10].

### Limitations

Our study had several inherent limitations given its retrospective design with a potential selection and information bias. However, we acquired and analyzed the complete data on the clinical outcomes of all patients. Second, although the hemodynamic data was not obtained through cardiac catheterization, we used Doppler-derived measurements that have been extensively validated to obtain a good correlation with the invasive hemodynamic indexes in the patients with DCM [30]. Third, in a subgroup analysis, we arbitrarily used the median value of the LVEDVI (≤ 160ml/m^2^ or >160ml/m^2^) as a cutoff point to stratify the degree of LV remodeling. Although it is well known that an increase in the LV volume reflects an increased LV wall stress and LV mass, leading to myocardial remodeling [28], there is no consensus on the optimal threshold of the LVEDV for quantification of LV remodeling in patients with NICM. Fourth, in this study, some patients were found to have an atypical LGE pattern for NICM (13 subendocardial and 29 transmural types), and these findings could suggest a previous history of spontaneous recanalization in myocardial infarction. However, we excluded the patients with evidence of significant coronary artery disease on angiogram before the CMR imaging. In addition, the incidence of an atypical LGE pattern in our study was similar to that in the previously published studies that have shown various LGE patterns in NICM patients with different etiologies [31–33]. Fifth, in the present study, LGE volume was quantified using a FWHM technique, not with the 2 standard deviations (SDs) method that has been proposed by official guidelines [34]. However, to date, the optimal method for LGE quantification remains controversial, and moreover, the FWHM technique has been also shown good reproducibility, as well as good agreement with the manual quantification [35]. Finally, the identification and quantification of interstitial fibrosis by a novel technique, such as T1 mapping was not available and the serial change in myocardial LGE was not assessed during the follow-up.

## Conclusions

First, the presence of LGE confined to the RVIP among NICM patients did not significantly increase the risk of adverse cardiac events. LGE limited to the RVIP was an indicator of a small LGE extent, with limited prognostic significance only in a selected subpopulation, particularly in patients with a less dilated LV. Furthermore, RVIP-LGE had a more favorable outcome than LV-LGE even with the same extent of the LGE, which strongly suggested the benign nature of LGE located in the RVIP. Second, a young age, female gender, elevated diastolic BP, presence of LBBB, and prolonged QTc interval were independent predictors of RVIP-LGE. Those variables may be associated with a small LGE extent, as well as the RVIP location. However, considering that the clinical impact of the RVIP-LGE could not be determined solely by the location or extent of the LGE, these variables may also sufficiently predict the RVIP-LGE. This unique LGE distribution may have an important prognostic implication in refining the current risk stratification for adverse cardiac events in NICM patients.

## Supporting information

S1 Fig **Kaplan-Meier event-free survival curve** for the composite endpoint among the 4 groups stratified according to the presence and location of the LGE in patients with an LVEDVI ≤ 160 ml/m^2^; LGE: late gadolinium enhancement; LVEDVI: left ventricular end diastolic volume index.
(TIF)

S1 Table **Cox proportional hazard analysis** for the predictors of a composite endpoint in a subgroup of patients with a LVEDVI ≤ 160 ml/m^2^; LVEDVI: left ventricular end diastolic volume index.
(DOCX)

## References

[1] Maron BJ, Towbin JA, Thiene G, Antzelevitch C, Corrado D, Arnett D, et al, Contemporary definitions and classification of the cardiomyopathies: an American Heart Association Scientific Statement from the Council on Clinical Cardiology, Heart Failure and Transplantation Committee; Quality of Care and Outcomes Research and Functional Genomics and Translational Biology Interdisciplinary Working Groups; and Council on Epidemiology and Prevention. Circulation. 2006; 113(14):1807–16. 10.1161/CIRCULATIONAHA.106.174287 . 16567565

[2] Rajappan K, Bellenger NG, Anderson L, Pennell DJ. The role of cardiovascular magnetic resonance in heart failure. Eur J Heart Fail. 2000; 2(3):241–52. . 10938483 10.1016/s1388-9842(00)00096-9

[3] Assomull RG, Prasad SK, Lyne J, Smith G, Burman ED, Khan M, et al Cardiovascular magnetic resonance, fibrosis, and prognosis in dilated cardiomyopathy. J Am Coll Cardiol. 2006; 48(10):1977–85. 10.1016/j.jacc.2006.07.049 . 17112987

[4] Gulati A, Jabbour A, Ismail TF, Guha K, Khwaja J, Raza S, et al Association of fibrosis with mortality and sudden cardiac death in patients with nonischemic dilated cardiomyopathy. JAMA. 2013; 309(9):896–908. 10.1001/jama.2013.1363 . 23462786

[5] Moon JC, McKenna WJ, McCrohon JA, Elliott PM, Smith GC, Pennell DJ. Toward clinical risk assessment in hypertrophic cardiomyopathy with gadolinium cardiovascular magnetic resonance. J Am Coll Cardiol. 2003; 41(9):1561–7. . 12742298 10.1016/s0735-1097(03)00189-x

[6] Bravo PE, Luo HC, Pozios I, Zimmerman SL, Corona-Vilalobos CP, Sorensen L, et al Late gadolinium enhancement confined to the right ventricular insertion points in hypertrophic cardiomyopathy: an intermediate stage phenotype? Eur Heart J Cardiovasc Imaging. 2016; 17(3): 293–300. 10.1093/ehjci/jev154 ; PubMed Central PMCID: PMC4750506. 26077330 PMC4750506

[7] Chan RH, Maron BJ, Olivotto I, Assenza GE, Haas TS, Lesser JR, et al Significance of Late Gadolinium Enhancement at Right Ventricular Attachment to Ventricular Septum in Patients With Hypertrophic Cardiomyopathy. Am J Cardiol. 2015; 116(3):436–41. 10.1016/j.amjcard.2015.04.060 . 26026863

[8] Kuribayashi T, Roberts WC. Myocardial disarray at junction of ventricular septum and left and right ventricular free walls in hypertrophic cardiomyopathy. Am J Cardiol. 1992; 70(15):1333–40. . 1442587 10.1016/0002-9149(92)90771-p

[9] Sato T, Tsujino I, Ohira H, Oyama-Manabe N, Nishimura M. Paradoxical motion of the interventricular septum as a primary mechanism of late gadolinium enhancement at ventricular insertion points. Int J Cardiol. 2012; 158(1):156–7. 10.1016/j.ijcard.2012.04.042 . 22560945

[10] Blyth KG, Groenning BA, Martin TN, Foster JE, Mark PB, Dargie HJ, et al Contrast enhanced-cardiovascular magnetic resonance imaging in patients with pulmonary hypertension. Eur Heart J. 2005; 26(19):1993–9. 10.1093/eurheartj/ehj328 . 15899927

[11] Freed BH, Gomberg-Maitland M, Chandra S, Mor-Avi V, Rich S, Archer SL, et al Late gadolinium enhancement cardiovascular magnetic resonance predicts clinical worsening in patients with pulmonary hypertension. J Cardiovasc Magn Reson. 2012; 14:11 10.1186/1532-429X-14-11 ; PubMed Central PMCID: PMC3311144. 22296860 PMC3311144

[12] Swift AJ, Rajaram S, Capener D, Elliot C, Condliffe R, Wild JM, et al LGE patterns in pulmonary hypertension do not impact overall mortality. JACC Cardiovasc Imaging. 2014; 7(12):1209–17. 10.1016/j.jcmg.2014.08.014 . 25496540

[13] Richardson P, McKenna W, Bristow M, Maisch B, Mautner B, O’Connell J, et al Report of the 1995 World Health Organization/International Society and Federation of Cardiology Task Force on the Definition and Classification of cardiomyopathies. Circulation. 1996; 93(5):841–2. . 8598070 10.1161/01.cir.93.5.841

[14] Felker GM, Shaw LK, O'Connor CM. A standardized definition of ischemic cardiomyopathy for use in clinical research. J Am Coll Cardiol. 2002; 39(2):210–18. . 11788209 10.1016/s0735-1097(01)01738-7

[15] Vrtovec B, Delgado R, Zewail A, Thomas CD, Richartz BM, Radovancevic B. Prolonged QTc interval and high B-type natriuretic peptide levels together predict mortality in patients with advanced heart failure. Circulation. 2003; 107(13):1764–9. 10.1161/01.CIR.0000057980.84624.95 . 12665499

[16] Lang RM, Badano LP, Mor-Avi V, Afilalo J, Armstrong A, Emande L, et al Recommendations for cardiac chamber quantification by echocardiography in adults: an update from the American Society of Echocardiography and the European Association of Cardiovascular Imaging. Eur Heart J Cardiovasc Imaging. 2015; 16(3):233–70. 10.1093/ehjci/jev014 . 25712077

[17] Messroghli DR, Radjenovic A, Kozerke S, Higgins DM, Sivananthan MU, Ridgway JP. Modified Look-Locker inversion recovery (MOLLI) for high-resolution T1 mapping of the heart. Magn Reson Med. 2004; 52(1):141–6. 10.1002/mrm.20110 . 15236377

[18] Cerqueira MD, Weissman NJ, Dilsizian V, Jacobs AK, Kaul S, Laskey WK, et al Standardized myocardial segmentation and nomenclature for tomographic imaging of the heart. A statement for healthcare professionals from the Cardiac Imaging Committee of the Council on Clinical Cardiology of the American Heart Association. Circulation. 2002; 105(4):539–42. . 11815441 10.1161/hc0402.102975

[19] Turkbey EB, Nacif MS, Noureldin RA, Sibley CT, Liu S, Lima JA, et al Differentiation of myocardial scar from potential pitfalls and artefacts in delayed enhancement MRI. Br J Radiol. 2012; 85 (1019):e1145–54. 10.1259/bjr/25893477 PMIC:23091294. 23091294 PMC3500815

[20] Cummings KW, Bhalla S, Javidan-Nejad C, Bierhals AJ, Gutierrez FR, Woodard PK. A pattern-based approach to assessment of delayed enhancement in nonischemic cardiomyopathy at MR imaging. Radiographics. 2009; 29(1):89–103. 10.1148/rg.291085052 . 19168838

[21] Neilan TG, Coelho-Filho OR, Danik SB, Shah RV, Dodson JA, Verdini DJ, et al CMR quantification of myocardial scar provides additive prognostic information in nonischemic cardiomyopathy. JACC Cardiovasc Imaging. 2013; 6(9): 944–54. 10.1016/j.jcmg.2013.05.013 ; PubMed Central PMCID: PMC3952043. 23932642 PMC3952043

[22] Gaztanaga J, Paruchuri V, Elias E, Wilner J, Islam S, Sawit S, et al Prognostic Value of Late Gadolinium Enhancement in Nonischemic Cardiomyopathy. Am J Cardiol. 2016; 118(7):1063–8. 10.1016/j.amjcard.2016.06.059 . 27614850

[23] de Leeuw N, Ruiter DJ, Balk AH, de Jonge N, Melchers WJ, Galama JM. Histopathologic findings in explanted heart tissue from patients with end-stage idiopathic dilated cardiomyopathy. Transplant. Int. 2001; 14(5):299–306. . 11692213 10.1007/s001470100339

[24] Mewton N, Liu CY, Croisille P, Bluemke D, Lima JA. Assessment of myocardial fibrosis with cardiovascular magnetic resonance. J Am Coll Cardiol. 2011; 57(8):891–903. 10.1016/j.jacc.2010.11.013 . 21329834 PMC3081658

[25] Malaty AN, Shah DJ, Abdelkarim AR, Nagueh SF. Relation of replacement fibrosis to left ventricular diastolic function in patients with dilated cardiomyopathy. J Am Soc Echocardiogr. 2011; 24(3):333–8. 10.1016/j.echo.2010.12.017 . 21338867

[26] Wilcox JE, Rosenberg J, Vallakati A, Gheorghiade M, Shah SJ. Usefulness of electrocardiographic QT interval to predict left ventricular diastolic dysfunction. Am J Cardiol. 2011; 108(12):1760–6. 10.1016/j.amjcard.2011.07.050 ; PubMed Central PMCID:PMC3637899. 21907948 PMC3637899

[27] Vanoverschelde JL, Raphael DA, Robert AR, Cosyns JR. Left ventricular filling in dilated cardiomyopathy: relation to functional class and hemodynamics. J Am Coll Cardiol. 1990; 15(6):1288–95. . 2329234 10.1016/s0735-1097(10)80016-6

[28] Alter P, Rupp H, Adams P, Stoll F, Figiel JH, Klose KJ, et al Occurrence of late gadolinium enhancement is associated with increased left ventricular wall stress and mass in patients with non-ischaemic dilated cardiomyopathy. Eur J Heart Fail. 2011; 13(9):937–44. 10.1093/eurhjf/hfr082 . 21803756

[29] Bogun FM, Desjardins B, Good E, Gupta S, Crawford T, Oral H, et al Delayed-enhanced magnetic resonance imaging in nonischemic cardiomyopathy: utility for identifying the ventricular arrhythmia substrate. J Am Coll Cardiol. 2009; 53(13):1138–45. 10.1016/j.jacc.2008.11.052 ; PubMed Central PMCID:PMC2747602. 19324259 PMC2747602

[30] Pozzoli M, Capomolla S, Pinna G, Cobelli F, Tavazzi L. Doppler echocardiography reliably predicts pulmonary artery wedge pressure in patients with chronic heart failure with and without mitral regurgitation. J Am Coll Cardiol. 1996; 27(4):883–93. 8613619 10.1016/0735-1097(95)00553-6

[31] McCrohon JA, Moon JC, Prasad SK, McKenna WJ, Lorenz CH, Coats AJ, et al Differentiation of heart failure related to dilated cardiomyopathy and coronary artery disease using gadolinium-enhanced cardiovascular magnetic resonance. Circulation. 2003; 108(1):54–9. 10.1161/01.CIR.0000078641.19365.4C . 12821550

[32] Almehmadi F, Joncas SX, Nevis I, Zahrani M, Bokhari M, Stirrat J, et al Prevalence of myocardial fibrosis patterns in patients with systolic dysfunction: prognostic significance of the prediction of sudden cardiac arrest or appropriate implantable cardiac defibrillator therapy. Circ Cardiovasc Imaging. 2014; 7(4):593–600. 10.1161/CIRCIMAGING.113.001768 . 24902587

[33] Perazzolo Marra M, De Lazzari M, Zorzi A, Migilore F, Zilio F, Calore C, et al Impact of the presence and amount of myocardial fibrosis by cardiac magnetic resonance on arrhythmic outcome and sudden cardiac death in nonischemic dilated cardiomyopathy. Heart Rhythm. 2014; 11(5):856–63. 10.1016/j.hrthm.2014.01.014 . 24440822

[34] Kramer CM, Barkhausen J, Flamm SD, Kim RJ, Nagel E; Society for Cardiovascular Magnetic Resonance Board of Trustees Task Force on Standardized Protocols. Standardized cardiovascular magnetic resonance imaging (CMR) protocols, society for cardiovascular magnetic resonance: board of trustees task force on standardized protocols. J Cardiovasc Magn Reson. 2008; 10:35 10.1186/1532-429X-10-35 . 18605997 PMC2467420

[35] Flett AS, Hasleton J, Cook C, Hausenloy D, Quarta G, Ariti C et al Evaluation of techniques for the quantification of myocardial scar of differing etiology using cardiac magnetic resonance. JACC Cardiovasc Imaging. 2011; 4(2):150–6. 10.1016/j.jcmg.2010.11.015 . 21329899

