# Supplementary figures and images for: Prognostic implications of late gadolinium enhancement at the right ventricular insertion point in patients with non-ischemic dilated cardiomyopathy: A multicenter retrospective cohort study
Source: PLoS One. 2018 Nov 28;13(11):e0208100. doi: 10.1371/journal.pone.0208100 (PMC6261623; doi:10.1371/journal.pone.0208100)

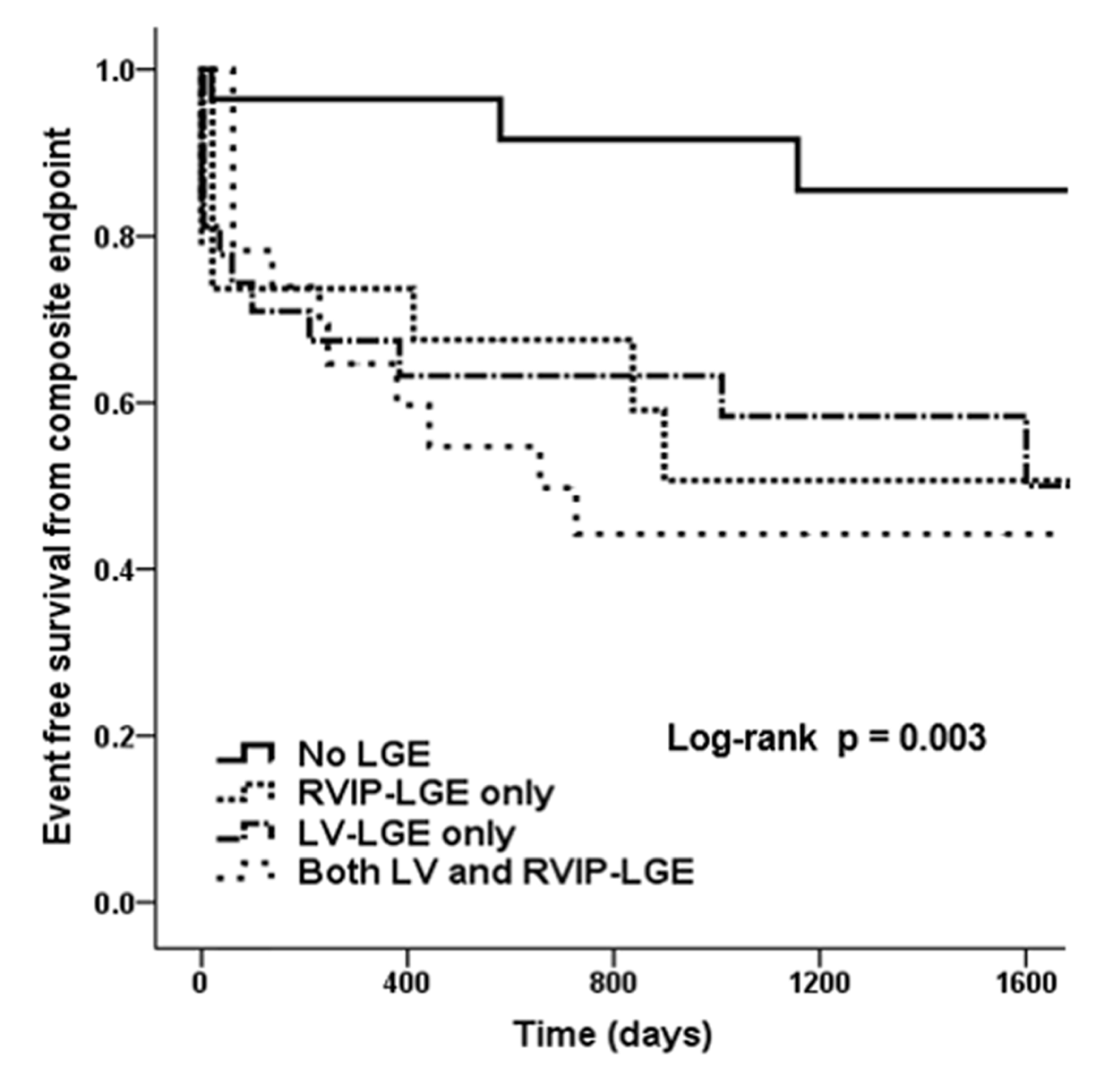

Supplement: S1 Fig — Kaplan-Meier event-free survival curve for the composite endpoint among the 4 groups stratified according to the presence and location of the LGE in patients with an LVEDVI ≤ 160 ml/m2; LGE: late gadolinium enhancement; LVEDVI: left ventricular end diastolic volume index. (TIF) [file pone.0208100.s001.tif]
