# Supplementary material for: Prognostic implications of late gadolinium enhancement at the right ventricular insertion point in patients with non-ischemic dilated cardiomyopathy: A multicenter retrospective cohort study
Source: PLoS One. 2018 Nov 28;13(11):e0208100. doi: 10.1371/journal.pone.0208100 (PMC6261623; doi:10.1371/journal.pone.0208100)
Supplement: S1 Table — Cox proportional hazard analysis for the predictors of a composite endpoint in a subgroup of patients with a LVEDVI ≤ 160 ml/m2; LVEDVI: left ventricular end diastolic volume index. (DOCX) [file pone.0208100.s002.docx]

**S1 Table 1 Cox proportional hazard analysis for the predictors of a composite endpoint in a subgroup of patients with a left ventricular end diastolic volume index ≤ 160ml/m^2^**

|  | **Univariate analysis** |  |  | **Multivariate analysis (χ2=30.701, p = 0.001)** |  |
| --- | --- | --- | --- | --- | --- |
|  | **HR (95% CI)** | ***p*** |  | **HR (95% CI)** | ***p*** |
| LGE location |  |  |  |  |  |
| No LGE | 1 (reference) |  |  | 1 (reference) |  |
| RVIP-LGE | 4.990 (1.526 ― 16.322) | 0.008 |  | 4.196 (1.213 ― 14.515) | 0.024 |
| LV-LGE | 5.729 (1.899 ― 17.286) | 0.002 |  | 4.028 (1.231 ― 13.176) | 0.021 |
| LV and RVIP-LGE | 7.089 (2.337 ― 21.501) | 0.001 |  | 4.382 (1.285 ― 14.945) | 0.018 |
| LGE extent (%) | 1.042 (1.021 ― 1.063) | <0.0001 |  | 1.042 (1.013 ― 1.071) | 0.004 |
| Age (years) | 1.009 (0.985 ― 1.033) | 0.476 |  | 1.009 (0.981 ― 1.038) | 0.536 |
| Female | 1.709 (0.945 ― 3.092) | 0.076 |  | 1.691 (0.732 ― 3.905) | 0.218 |
| NYHA class ≥ III | 1.174 (0.641 ― 2.150) | 0.604 |  | 1.125 (0.567 ― 2.232) | 0.736 |
| Diabetes mellitus | 1.345 (0.741 ― 2.440) | 0.329 |  | 1.601 (0.827 ― 3.100) | 0.162 |
| Smoking | 0.841 (0.441 ― 1.602) | 0.598 |  | 0.776 (0.324 ― 1.856) | 0.568 |
| LVEF (%) | 0.997 (0.962 ― 1.033) | 0.850 |  | 0.968 (0.928 ― 1.011) | 0.143 |

HR, hazard ratio; CI, confidence interval; LGE, late gadolinium enhancement; RVIP, right ventricular insertion point; NYHA, New York Heart Association; LVEF, left ventricular ejection fraction.
